# Perception on the severity of unwanted pregnancy among university students

**DOI:** 10.12669/pjms.294.3626

**Published:** 2013

**Authors:** Felix Chima Anyanwu, Daniel Ter Goon, Augustine Tugli

**Affiliations:** 1 Felix Chima Anyanwu, Department of Public Health, University of Venda, Thohoyandou, South Africa.; 2 Daniel Ter Goon, Centre for Bioknetics, Recreation and Sport Science, University of Venda, Thohoyandou, South Africa.; 3 Augustine Tugli, Department of Public Health, University of Venda, Thohoyandou, South Africa.

**Keywords:** Unwanted pregnancy, Perception, University students

## Abstract

***Objectives:*** The purpose of this study was to examine the perception of University students regarding the severity of unwanted pregnancy.

***Methods:*** This cross sectional study involved 408 (206 females and 202 males) students residing within the university campus. Simple and systematic sampling methods were used to select participants. A 4-likert scaled self-administered questionnaire was used for data collection.

***Result:*** Majority (87.70%) of participants perceived unwanted pregnancy as leading to impaired mental health; 86.30% perceived it as a cause of many other health problems; 86.60% believed it could result to shame and withdrawal from society or even suicidal attempts; and child neglect and abandonment (84.80%). Using the cut-off points of 75% of the total scores as a criteria for assessing perception, fewer (60.30%) participants perceived unwanted pregnancy as preventing a girl from continuing with her education; insufficient money to provide for both mother and child (74.50%) and leading to higher risk of substance abuse and problem behaviour among children born from unwanted pregnancies (51.20%). Females students strongly agreed that unwanted pregnancy could lead to shame and withdrawal from the society compared to their male counterparts (Chi-square = 10.788, p = 0.013).

***Conclusion:*** Few students at the University of Venda perceived unwanted pregnancy as being severe enough and associated with truncated education, poverty for the young mother, and increased risk of problem behaviours. Thus, intervention strategies should be instituted to prevent unwanted pregnancies among the students.

## INTRODUCTION

Young people are at increased risk for unplanned pregnancy, and the highest rate of unwanted pregnancy has been reported among individuals between the ages of 14–19 years.^1^ In sub-Saharan Africa, it is estimated that 14 million unwanted pregnancies occur every year, with almost half occurring among women aged 15–24 years.^2^

In Japan, only 36% of child births are intended^3^ while in Turkey, the Population and Health Research unit estimates that 20% of all birth between 1998 and 2003 were unwanted and 14% were unplanned.^4^ In the USA, almost half of pregnancies are unplanned,^5^^-^^6^ with a prevalence of about 63% among African American women and women with low income.^7^ One out of every three pregnancy ending in a live birth are unwanted^8^ and about 40% of young women become pregnant before reaching 20 years of age.^9^ Although, there are claims that unintended pregnancy rate has declined by 29 percent in developed regions and by 20 percent in developing regions,^10^ the number of adolescent pregnancies in South Africa has continued to rise despite efforts by the government to make contraception available to all.^11^

In developing countries, young women have a range of factors that limits their use of modern contraception, these factors are lack of knowledge, obstacle to access and concern over side effects.^2^ In addition, premarital exposure to pregnancy risk has increased, with a widening gap between the age at first sexual experience and the age of marriage, giving rise to increased sexual activity before marriage.^2^

There is a relationship between an unwanted pregnancy and poor health outcome for the child^3^^,^^12^ and society^13^ as shown by the estimated overall cost of between 7 and 15 billion dollars spent on teenage childbearing in the United States of America (USA) annually.^13^ It has been associated with poor prenatal behaviour, as women in this condition may not recognise that they are pregnant on time, they have also been shown to delay initiations of prenatal care and also engage in substance abuse during pregnancy^3^ and it is also associated with low economic status and termination of educational career for the young mother.^14^ Five million out of the estimated 20 million cases of unsafe abortions that occur each year and about 70,000 abortion related deaths annually occur among women aged 15–19 years and this age group is twice as likely to die during childbirth as women aged 20 years and above.^2^ It is also estimated that 90% of abortion-related and 20% of pregnancy-related morbidity and mortality along with 32% of maternal deaths could be prevented if effective contraception is used at the time of sexual intercourse.^2^

It has been shown that only those who perceive pregnancy and childbearing negatively would regard contraception in a positive manner and adopt a consistent protective lifestyle.^15^ This study therefore, aims to determine the perception of university students regarding the severity of an unwanted pregnancy.

## METHODS

Simple and systematic sampling methods were used to select the rooms from which participants were chosen. Each cluster (residence hall) constituted a proportion of the total population depending on the number of bed spaces available to it. This was done to ensure accurate representation of each residence. Every 6th room was selected for the study. The room numbers on each block was written on a sheet of paper and placed in an envelope from which the first room was randomly selected and subsequent rooms were selected after every 6^th^ room.

A questionnaire was used for data collection. It was divided into two sections. Section A solicits the demographic profile of the respondents while Section B provides background information on sexual experiences. The reliability of the instrument was measured using the test-retest method and the correlation coefficient was calculated to be 0.82 which is within acceptable limits.^16^

Permission to conduct the research was given by the University of Venda, health, safety and research ethics committee: SHS/12/PH/06/0912. The purpose and nature of the study was explained to the participants. Questionnaires were given to those who signed the consent form and they also received A4 sized envelopes so that the completed questionnaire can be sealed off and deposited at the security post for collection by the research team. 

Four hundred and fifteen questionnaires were distributed, and a return rate of 69.17%. Seven questionnaires were not properly completed and were excluded from the study, leaving 408 questionnaires for analysis. 

Data was analyzed with Statistical Package for Social Sciences (SPSS). Chi-square test was used to compare differences between variables and statistical difference was set at p<0.05.

## RESULTS

Participants mean age was 22.20±3.53. More than half of the study participants (59.10%) were between 20 to 24 years and gender distribution was almost equal with 50.49% female and 49.51% male. Three hundred and ninety seven participants (97.30%) were Christians, most were undergraduates (93.60%) and single (97.50%). Two hundred and thirteen (52.20%) reported living with both of their parents and 195 (47.80%) reported living with either parent.

**Fig.1:**
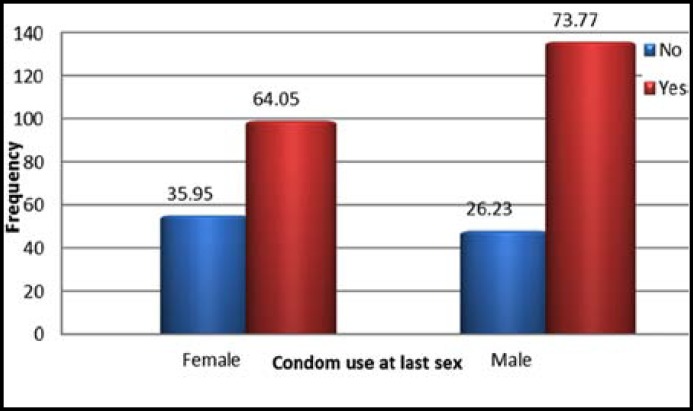
The rate of condom use at last sexual intercourse

Among the sexually active participants, 135 (73.77% of sexually active males) reported using condom at the last sex while 98 (64.05% of sexually active females) reported not using condom at last sex (Fig.1).

Three hundred and eighty five (87.70%) participants either agreed or strongly agreed that an unwanted pregnancy could result to impaired mental health while 50 (12.30%) participants either disagreed or strongly disagreed. On whether pregnancy could prevent a girl from continuing with her education, 246 (60.30%) participants either agreed or strongly agreed while 162 (39.70%) either disagreed or strongly disagreed. Majority (86.3%) of the participants either agreed or strongly agreed that unwanted pregnancy could lead to many health problems, while 80.60% agreed or strongly agreed that it could lead to shame, withdrawal or even suicidal attempts (Table-I).

**Table-I:** Perceived severity of the negative outcomes associated with unprotected sex

*Statements on unwanted pregnancy*	*n (%)*			
	*SA*	*A*	*D*	*SD*
It may cause impaired mental health	202(49.50)	156(38.20)	26(6.40)	24(5.90)
It could lead to school dropout.	100(24.50)	146(35.80)	111(27.20)	51(12.5)
It could lead to other health problem	181(44.40)	171(41.90)	44(10.80)	12(2.90)
May lead to shame and withdrawal from society or even suicidal attempt	143(35.00)	186(45.60)	56(13.70)	23(5.60)
No money to take care of baby	151(37.00)	153(37.50)	72(17.60)	32(7.80)
May lead to child neglect	187(45.80)	159(39.00)	43(10.50)	(194.70)
HIV infection may cause devastation and neglect of responsibility	208(51.00)	147(36.00)	32(7.80)	21(5.10)
HIV/AIDS may result in drop out of school	135(33.10)	167(40.90)	72(17.60)	34(8.30)
STI may lead to infertility	117(28.70)	181(44.40)	75(18.40)	35(8.60)
Babies from unwanted pregnancies are more likely to abuse substances	91(22.30)	108(26.50)	102(25.00)	107(26.20)

SA = Strongly Agree; A = Agree; D = Disagree; SD = Strongly Disagree

On whether there will be enough money to take care of a baby when the pregnancy was not wanted, 304 (74.50%) participants agreed or strongly agreed that financial support might be a problem while 104 (24.50%) disagreed or strongly disagreed. Regarding the issue of child neglect and abandonment, 346 (84.80%) participants either agreed or strongly agreed that an unwanted pregnancy may lead to a situation whereby a baby may be neglected or even abandoned. Three hundred and fifty five (87.00%) participants agreed or strongly agreed that one could be devastated and therefore neglect their responsibility when they find out that they have been infected with HIV after exposing themselves to unprotected sex .Two hundred and nine participants (51.20%) either disagreed or strongly disagreed that babies resulting from an unwanted pregnancy are more likely to abuse substances and have problem behaviours than those whose pregnancies were planned.

There was no significant difference in the way males and female participants perceive the effects of unwanted pregnancy on mental health (Chi-square = 5.705, p = 0.127). There was no significant difference found on whether unwanted pregnancy could prevent a girl from continuing her education (Chi-square = 1.284, p = 0.733) or whether complications resulting from unprotected sex may result in drop out of school (Chi-square = 0.522, p-value 0.914) (Table-II).

**Table-II:** Gender and perceived health complications and there effects on education

*Statements*		*Female*		*Male*		*Total*		
		*n*	*%*	*n*	*%*	*n*	*%*	
When a young girl gets pregnant, it could prevent her from continuing with her education.	SA	54	26.21	46	22.77	100	24.51	Chi-square 1.284; p-value 0.733
	A	69	33.50	77	38.12	146	35.78	
	D	58	28.16	53	26.24	111	27.21	
	SD	25	12.14	26	12.87	51	12.50	
Complications resulting from unprotected sex (eg HIV/AIDS) may result in drop out of school	SA	68	33.01	67	33.17	135	33.09	Chi-square 0.522; p-value 0.914
	A	82	39.81	85	42.08	167	40.93	
	D	39	18.93	33	16.34	72	17.65	
	SD	17	8.25	17	8.42	34	8.34	

SA = Strongly Agree; A = Agree; D = Disagree; SD = Strongly Disagree

There was a significant difference between male and female on whether unwanted pregnancy could lead to shame and withdrawal from the society (Chi-square = 10.788, p = 0.013) (Table-III).

**Table-III:** Gender and perceived complications resulting from unprotected sex

*Statements*		*Female*		*Male*		*Total*		
		*n*	*%*	*n*	*%*	*n*	*%*	
Unwanted pregnancy may lead to shame and withdrawal from the society or even suicidal attempt	SA	86	41.75	57	28.22	143	35.05	Chi-square;10.788 p-value 0.013
	A	86	41.75	100	49.50	186	45.59	
	D	21	10.19	35	17.33	56	13.76	
	SD	13	6.31	10	4.95	23	5.64	
When a person is infected with HIV, it could result in devastation and neglect of responsibility	SA	104	50.49	104	51.49	208	50.98	Chi-square 0.140;p-value 0.987
	A	74	35.92	73	36.14	147	36.03	
	D	17	8.25	15	7.43	32	7.85	
	SD	11	5.34	10	4.95	21	5.15	

SA = Strongly Agree; A = Agree; D = Disagree; SD = Strongly Disagree

There was no significant difference found on whether an unwanted pregnancy could lead to child neglect and abandonment (Chi-square = 1.509, p = 0.680) and if there would be enough money to take care of the baby (Chi-square = 2.992, p = 0.393) (Table-IV).

**Table-IV:** Gender and perceived consequences for the Child

***Statements***		***Female***		***Male***		***Total***		
		***n***	***%***	***n***	***%***	***n***	***%***	
When a pregnancy is not wanted, there might not be enough money to take care of the baby	SA	81	39.32	70	34.65	151	37.01	Chi-square 2.992 p-value 0.393
	A	70	33.98	83	41.09	153	37.50	
	D	36	17.48	36	17.82	72	17.64	
	SD	19	9.22	13	6.44	32	7.85	
Unwanted pregnancy may lead to child neglect or abandonment	SA	95	46.12	92	45.54	187	45.85	Chi-square 1.509; p-value 0.680
	A	83	40.29	76	37.62	159	38.97	
	D	18	8.74	25	12.38	43	10.54	
	SD	10	4.85	9	4.46	19	4.66	
Babies from unwanted pregnancies are more likely to abuse substances	SA	41	19.90	50	24.75	91	22.30	Chi-square 3.779; p-value 0.286
	A	51	24.76	57	28.22	108	26.47	
	D	59	28.64	43	21.29	102	25.00	
	SD	55	26.70	52	25.74	107	26.23	

SA = Strongly Agree; A = Agree; D = Disagree; SD = Strongly Disagree

Among the sexually active participants who did not use condom at their last sexual intercourse, 90 (87.38%) participants either agree or strongly agree that someone could be devastated and even neglect his or her responsibility after they discover they are HIV positive while 13 (12.62%) either disagreed or strongly disagreed. However, there was no association between condom use at last sex and the perception that when a person is infected with HIV, it could result in devastation and neglect of responsibility.

## DISCUSSION

The result of this study shows age range between 15 to 45 years with a mean age of 22.20 years (SD = 3.53). This is comparable to age profile among university students in KwaZulu Natal^17^ and Lesotho.^18^ The study indicated that majority of the students believed that an unwanted pregnancy may result to impaired mental health and other health problems; indicating no significant gender difference. This finding is similar to a study which found that women with unplanned pregnancy had fewer positive prenatal care behaviours, experienced more physical problems, had more negative experiences and pain in labour, and experienced more mental problems in the early postpartum period.^19^

Although studies have shown that young mothers tend to have fewer years of education when compared to those who have their first child after 20 years of age.^20^ The impart of unwanted pregnancy on the education of a young person depends on the timing of the pregnancy and the outlook of the girl and her family regarding unwanted pregnancy.^21^ In addition, only about a third of young women in South Africa return to school after pregnancy^22^ and less than two third (60.30%) of the students in this present study believed that the scholastic ambition of a young girl could be truncated as a result of an unwanted pregnancy. This perception was low and showed no significant difference between male and female. However, it was slightly lower than that reported in a Nigerian university; showing that 62% of participants agreed that unwanted pregnancy could cause termination of educational career.^14^

The perception that an unwanted pregnancy could lead to shame and withdrawal from the society was high with an associated gender bias (females agreeing more). This perception was much higher than that described in a Nigerian University^14^ where only 33% reported that unwanted pregnancy could lead to shame and withdrawal from the society. The stigma associated with pregnancy at an early age is high and girls face fear, shame and embarrassment when revealing an early pregnancy to family, partners and peers and this could result to depression, social exclusion, and low self-esteem.^23^

The financial implication of pregnancy is high; it is even higher for a young mother and especially when the pregnancy is unwanted. Almost three quarter (74.50%) of the students perceived an unwanted pregnancy as causing financial constraints on the young mother (there was no significant difference in gender perception). This figure is lower than that described in the University of Zululand, where 97% of participants agreed that unwanted pregnancy could lead to poverty for the girl and her child and could further cause financial strain on the family income as they would have to take care of her and the baby since she is not working ^24^ and also lower than 95.8% described in a Nigerian university.^14^ It was widely believed among participants in the present study that child neglect and abandonment may result following an unwanted pregnancy.

Academic knowledge has a minimal impact on health related behaviour; and young people take risk even when they know that such behaviours endanger their lives.^25^ The present study agrees with this statement as majority (87.38%) of the students who did not use condom at last sex also believe that someone could be devastated and even neglect his or her responsibility after they discover they are HIV positive.

## CONCLUSION

The students in this present study perceived that an unwanted pregnancy could have health implications for the young mother and could result to shame, withdrawal from the society, suicidal attempts, child neglect and abandonment. Only few believed that unwanted pregnancy might truncate their educational career. 

## Authors’ contributions

**FCA **was the primary investigator for the study, designed the study, supervised data collection and wrote the paper.

**DTG** advised on data collection and helped write the paper.

**AT** participated in the analysis of the findings and writing the paper. All authors participated in writing of the manuscript, read and approved the final manuscript.
